# Fluxional Halogen Bonds Versus Interlayer Stacking – Theory Meets Experiment

**DOI:** 10.1002/chem.202502514

**Published:** 2025-11-07

**Authors:** Cai Yue Gao, Annika Schmidt, Ruimin Wang, Carsten Strohmann, Ulli Englert, Si‐Dian Li

**Affiliations:** ^1^ Institute of Molecular Science Shanxi University Taiyuan 030006 China; ^2^ Institute of Inorganic Chemistry TU Dortmund University 44227 Dortmund Germany; ^3^ Institute of Inorganic Chemistry RWTH Aachen University 52056 Aachen Germany; ^4^ Institute of Environmental and Chemical Engineering Jiangsu University of Science and Technology Zhenjiang 212003 China

**Keywords:** crystal‐to‐crystal transition, experimental electron density, halogen bonds, revised van der Waals density functional theory

## Abstract

Halogen bonds (XBs) represent strongly directional interactions, relevant for bond activation and often quoted in the context of Crystal Engineering. Bifurcated XBs, albeit rare, are unambiguously established in crystalline solids but do not correspond to an energetically favorable arrangement at the molecular level. Here the dynamic behavior of such a system, jointly described by experiment and theory, reconciles both aspects: Experiment shows that the cocrystal of 1,4‐diiodobenzene and 1,4‐dinitrobenzene with a symmetric bifurcated XB undergoes a rarely observed reversible crystal‐to‐crystal transition at 130 K. The resulting low‐ temperature phase features an asymmetric bifurcated XB, a first step toward a more linear arrangement. Theoretical calculations based on revised van der Waals density functional theory and diffraction experiments conducted at high resolution agree remarkably well in their interpretation of transition temperature and relevance of interaction types. The XB is fluxional and entropy‐favored in the symmetric high‐ temperature phase and locks into an asymmetric geometry with slightly more favorable Gibbs free energy at low temperature; the energy differences involved are extremely small. Theoretically derived and experimentally observed electron densities agree that the perceived directional interactions are weak rather than decisive synthons. Stacking of alternating diiodobenzene and dinitrobenzene molecules represents the most relevant contact.

## Graphical abstract

Joint high‐resolution X‐ray diffraction and density functional theory investigations reveal the existence of fluxional halogen bonds in the cocrystal of 1,4‐diiodobenzene and 1,4‐dinitrobenzene. At 130 K this solid undergoes a reversible crystal‐to‐crystal phase transition from the high‐temperature **1**α phase with a symmetric bifurcated halogen bond to a low‐temperature twin **1**β with an asymmetric bifurcated halogen bond.

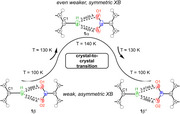

## 1 Introduction

**Figure 1:**
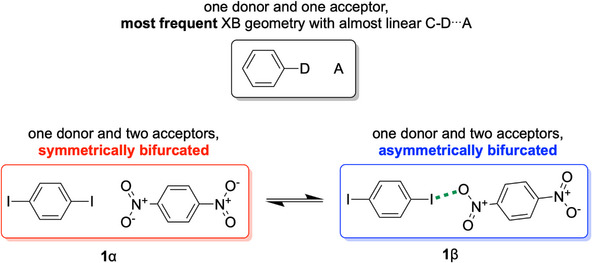
Halogen bond geometries relevant for this article. Top: most popular linear arrangement. Bottom left: symmetric bifurcated XB as described by Allen et al. in ref. [24]. Bottom right: asymmetric bifurcated XB, low temperature phase (this work); the slightly shorter contact has been emphasized by a dashed green line.

Crystal structures are often regarded as the authoritative answer concerning the spatial arrangement of atoms, not only with respect to the geometry of individual molecules but also in discussions about the relevance of intermolecular interactions. The Cambridge Structural Database (CSD)^[^ ^1^ ^]^ allows to screen a plethora of diffraction results for preferred arrangements of certain functional groups. This wealth of information is mostly exploited in a statistical manner; obviously, we here cannot provide a full survey of successful approaches. The very idea of *Crystal Engineering* is closely associated with the concept of such “supramolecular synthons.”^[^ ^2^ ^]^ Next to hydrogen bonds (HBs),^[^ ^3^, ^4^ ^]^ halogen bonds (XBs)^[^ ^5^ ^]^ count among the apparently easy to spot and directional interactions. The most general definition of an XB assumes “evidence of a net attractive interaction between an electrophilic region associated with a halogen atom in a molecular entity and a nucleophilic region in another, or the same, molecular entity.”^[^ ^6^ ^]^ In structural chemistry, an XB often shows up as a short contact between the σ hole^[^ ^7^ ^]^ of a (mostly heavy) halogen atom D and a lone pair of a suitable XB acceptor atom A; an example geometry for such an XB is depicted in Figure 1, top. XBs do not only represent often‐quoted interactions in fields such as *Crystal Engineering*; rather, the associated concept of bond activation has proven useful in organic synthesis and organocatalysis.^[^ ^8^, ^9^, ^10^, ^11^, ^12^ ^]^ The relevance of XBs has also been recognized in biological systems.^[^ ^13^, ^14^, ^15^ ^]^

**Figure 2:**
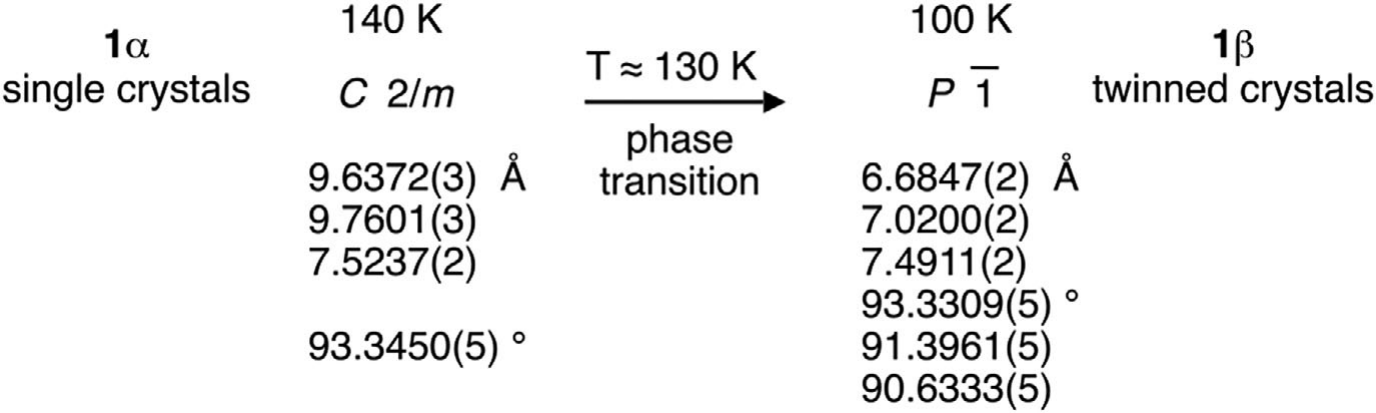
Symmetry and lattice parameters for crystals of 1,4‐dinitrobenzene/1,4‐diiodobenzene above and below the phase transition temperature.

In view of their importance both for basic research and application, it is not surprising that XBs have been studied extensively. The electronic situation in XB interactions may be addressed by theoretical calculations and can in favorable cases be verified by experimental electron density studies based on high resolution X‐ray diffraction.^[^ ^16^, ^17^, ^18^, ^19^, ^20^, ^21^, ^22^ ^]^ Both XBs and HBs are considered directional, but in contrast to the situation with HBs, bifurcation appears to be rather rare for XBs. Michalczyk, Zierkiewicz, and Scheiner have undertaken a detailed study of XBs in the CSD, together with a comparison to model systems.^[^ ^23^ ^]^ These authors have found that bifurcated XBs occur with a frequency lower than 2%. Long before this systematic study and following earlier ideas of crystal engineering, Allen et al.^[^ ^24^ ^]^ synthesized and structurally characterized the cocrystal of 1,4‐diiodobenzene with 1,4‐dinitrobenzene (**1**α in Figure 1) and thus obtained an early example for a completely symmetric bifurcated XB. Ji et al. theoretically proposed the existence of complexes featuring symmetric bifurcated halogen bonding interactions, which were subsequently verified through successful crystal synthesis.^[^ ^25^ ^]^ More recently, Jain and colleagues achieved the synthesis of a novel ternary crystalline compound; it was found to exhibit similar symmetric bifurcated XBs.^[^ ^26^ ^]^ Inspired by the discrepancy between bifurcation and the rather popular linear σ hole interactions, some of us have recently investigated the analogous molecular systems involving 1,4‐dinitrobenzene and 1,4‐diiodoarenes, namely the weak XB donor 1,4‐diiodobenzene as well as the F‐substituted stronger XB donor 1,4‐diiodotetrafluorobenzene.^[^ ^27^ ^]^ The results of this study suggest a dynamical situation: short strands of a few interacting XB donors and acceptors interact via fluxional XBs. For aggregates involving only a small number of molecules, the fully symmetric bifurcated XB may be perceived as transition state (TS) between two degenerate structural minima. These energy minima feature linear XBs between the iodine donor and the alternative oxygen acceptors of the nitro group; libration of the rigid arenes switches from one energetically favorable linear XB via the bifurcated arrangement to the alternative linear XB. These results from theoretical calculations match the observed solid‐state preference for linear over bifurcated XBs and suggest that a team of theoreticians and experimentalists should jointly tackle the alternative XB arrangements. Structure correlation theory^[^ ^28^ ^]^ assumes that (small) energy differences should be reflected in the frequencies in which crystal structures are encountered. In this contribution, we provide experimental and theoretical results which support the idea of fluxional XBs. We revisit the 1,4‐diiodobenzene/1,4‐dinitrobenzene cocrystal and show that the structure described by Allen et al.^[^ ^24^ ^]^ represents the high‐ temperature phase of a dynamical system. At 130 K, a rarely observed and fully reversible crystal‐to‐crystal transition occurs. This second‐order structural phase transition leads to an energetically more favorable solid with lower symmetry (**1**β in Figure 2). In the closer‐packed low temperature form, the onset of distortion from a symmetric to an asymmetric but more effective XB is observed, a first step on the path between a symmetric bifurcated XB and the more popular linear arrangement. The excellent quality of crystals before the phase transition allowed for high resolution diffraction and determination of an experimentally derived electron density, suitable for direct comparison with results from theory.

## 2 Results and Discussion

### 2.1 Experimental Observations

Cocrystals of 1,4‐dinitrobenzene/1,4‐diiodobenzene could be grown from ethanol and acetone (Supporting Information). Suitable crystals of good quality were studied by X‐ray diffraction; based on intensity data obtained at ambient temperature and at 140 K, we can fully confirm the earlier report by Allen et al.^[^ ^24^ ^]^ Upon further decrease in temperature, however, the diffraction pattern became more complex; as the result of a structural phase transition, twinned crystals had formed. Figure 2 summarizes the relationship between the previously reported high temperature phase **1**α and the new low temperature phase **1**β. Further details concerning the symmetry relationship between the phases^[^ ^29^ ^]^ and the evolution of the structures as a function of temperature have been compiled in the Supporting Information.

**Figure 3:**
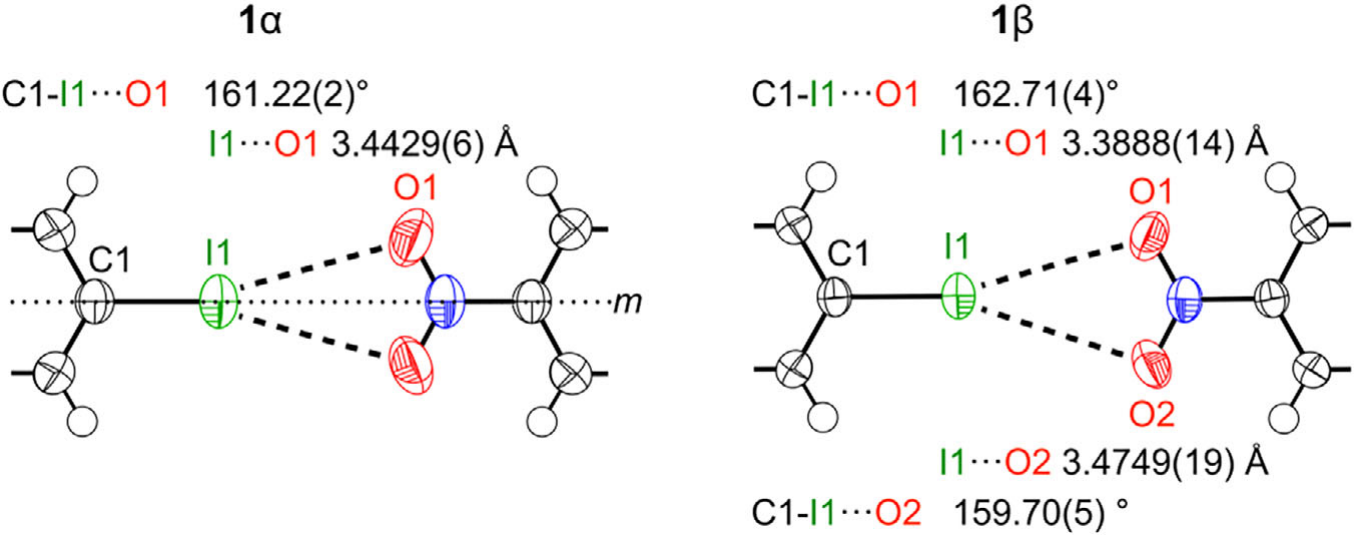
Fully symmetric bifurcated XB in the high‐temperature phase and slightly nonsymmetric XB in the low‐temperature phase; *m* denotes the crystallographic mirror plane in **1**α; displacement ellipsoids drawn at 90% probability.

The **1**α↔**1**β phase transition described in Figure 2 is fully reversible, and the diffraction pattern of the twinned crystals **1**β still allowed for a highly accurate structure determination (Supporting Information). Our first focus concerns the structural changes in the vicinity of the bifurcated XB. Figure 3 summarizes the geometry in this region of both high‐ and low‐temperature phases.

In **1**α, the contact distances between I and both O atoms in the closest nitro group are equivalent by symmetry. In **1**β, this equivalence is lifted; slightly but significantly different interaction distances and angles are observed. This low temperature geometry may be perceived as a first step toward a presumably shorter and more linear XB C1─I1···O1 and a longer contact between I1 and O2. Based on the geometry in both phases, we already note that the I···O distances are only slightly shorter than the sum of the van‐der‐Waals radii^[^ ^30^ ^]^ and considerably longer than those commonly associated with XBs.^[^ ^17^, ^22^, ^31^ ^]^

**Figure 4:**
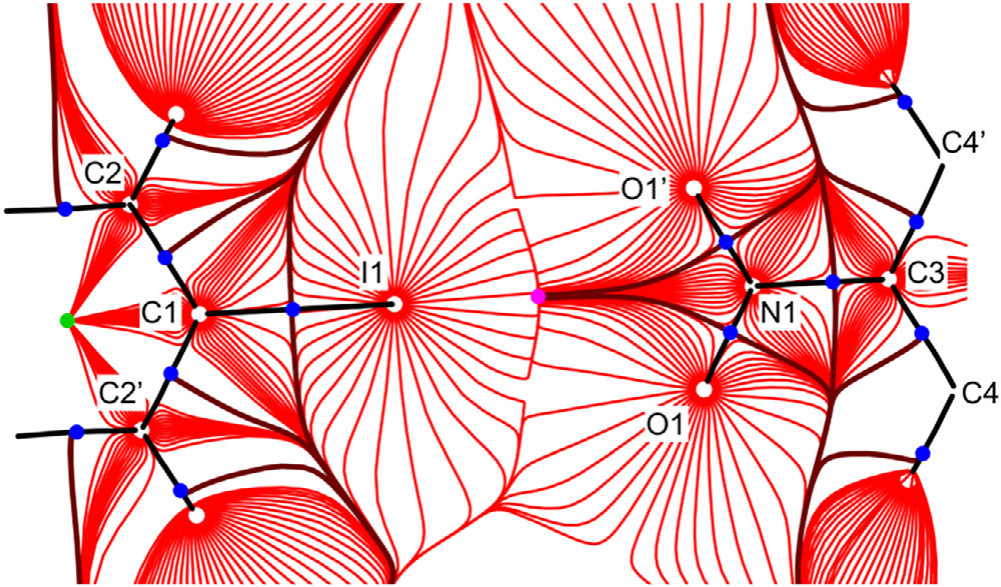
Gradient vector field of the experimental electron density; bond paths are shown as black lines. Nuclear attractors are depicted as white, bcps as dark blue, ring critical points as green, and cage critical points as magenta solid circles. Primed atoms are related to unprimed ones by the symmetry operation *x, −y, z*.

The excellent quality of the crystals allowed for diffraction experiments up to very high resolution (see Supporting Information), with two relevant consequences. On the one hand, the derived geometry data are associated with small standard uncertainties, and even subtle structural changes may reliably be addressed. On the other hand, data for the (untwinned) **1**α phase meet the requirements for a multipole refinement^[^ ^32^, ^33^ ^]^ and an experimental description of the electron density in the crystal. All details concerning data completeness and refinement have been compiled in the Supporting Information; we here only report the most relevant results. The experimental electron density may be analyzed following Bader's Quantum Theory of Atoms in Molecules (QTAIM)^[^ ^34^ ^]^ approach. With the help of QTAIM, extrema and saddle points of the experimental electron density can be interpreted. Atoms are delimited by their zero flux surfaces. Intramolecular bonds as well as sufficiently strong intermolecular contacts are associated with a bond path and saddle points of the electron density, so‐called bond critical points (bcps). From the electron density in such bcps, energy densities can be calculated and conclusions concerning the nature and the strength of the interactions can be drawn. Figure 4 shows a trajectory plot of the electron density in the plane subtended by iodine and the oxygen atoms of the nitro group, the region most relevant for the bifurcated XB.

Figure 4 reveals the expected features of the electron density for the covalent bonds in the residues dinitrobenzene and diiodobenzene; all are associated with a bond path and a bond critical point. No bcp between the iodine atom and the closest nitro group is detected, however; the bond paths rather collapse to a local density minimum, a cage critical point. In previous studies, we have been able to detect even those bcps which are associated with rather low electron densities typically encountered in weak XBs.^[^ ^20^, ^21^ ^]^ The complete absence of a bcp reliably indicates that the I···O contacts in the bifurcated XB, although originally believed to represent a synthon for crystal engineering, in reality are very weak.

**Figure 5:**
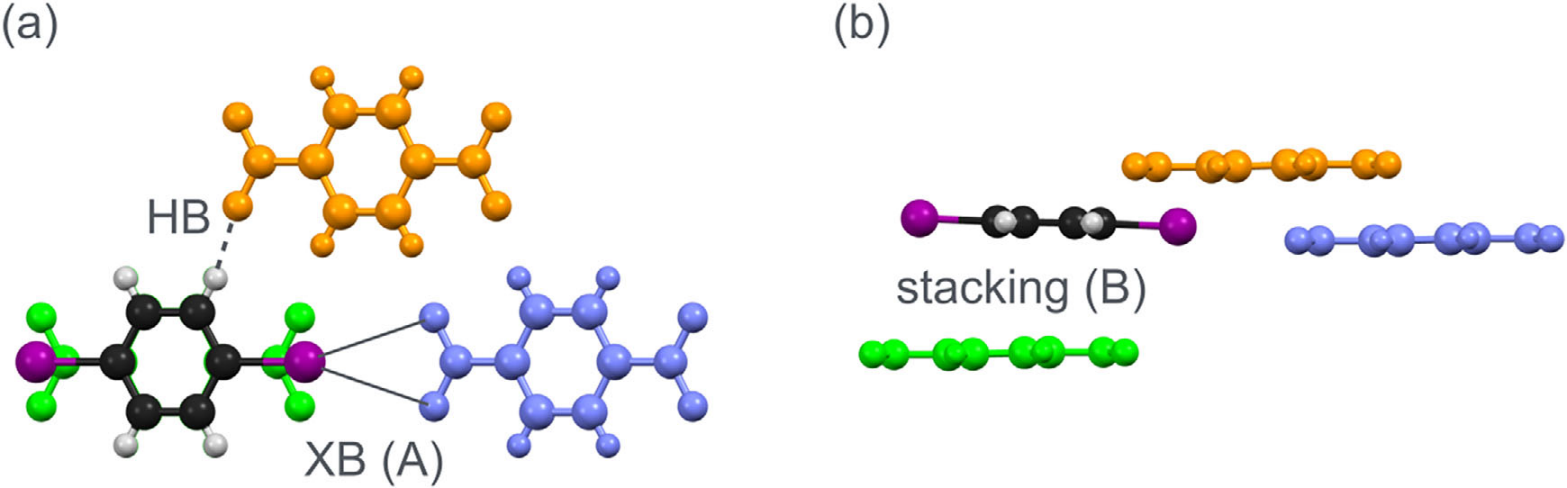
Short contacts in **1**α. A 1,4‐diiodobenzene molecule and three 1,4‐dinitrobenzene neighbors are shown; view directions are [0 0 1] in part (a) and [0 1 0] in part (b) of the figure. a) The 1,4‐dinitrobenzene depicted in light blue acts as XB acceptor (pattern A), the one depicted in orange as HB acceptor. b) 1,4‐diiodobenzene and its dinitrobenzene neighbor shown in light green are stacked along [0 0 1]; the shortest interatomic distance between stacked molecules in this pattern B amounts to 3.45 Å.

The bifurcated XB apart, Allen et al. also discuss another type of secondary interactions in **1**α, namely C─H···O and C─H···I contacts. A summary of the short contacts between a molecule of diiodobenzene and its next neighbors in crystalline **1**α is given in Figure 5.

**Figure 6:**
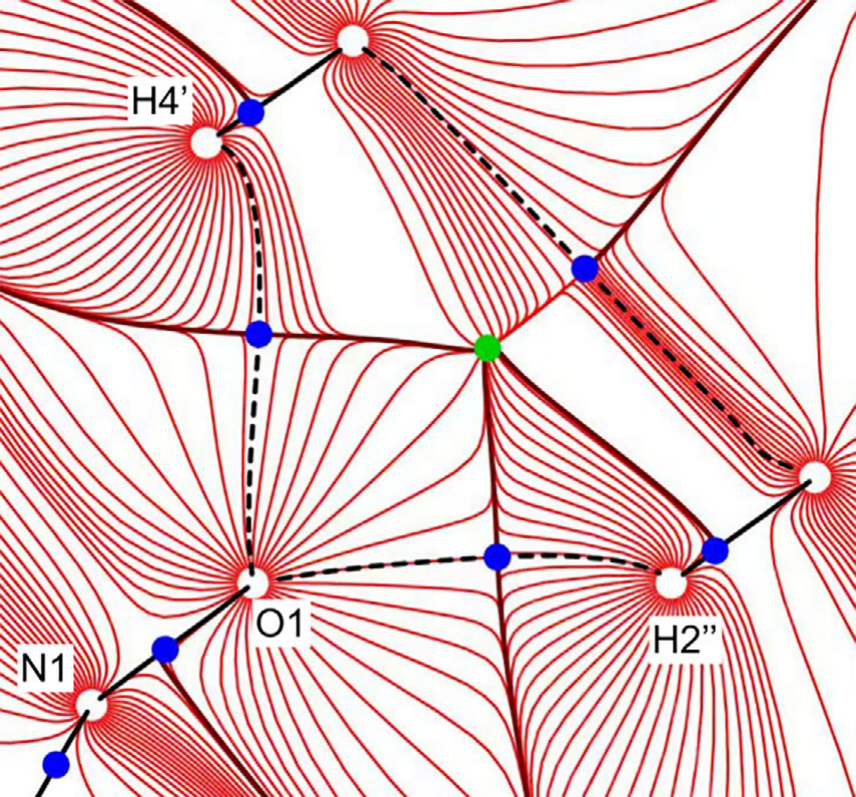
Gradient vector field of the experimental electron density in the plane subtended by O1, H2‘’ (symmetry operation −0.5 + x, −0.5 + y, z), and H4' (symmetry operation 0.5−x, −0.5−y, −z). Bond paths are shown as black lines, nuclear attractors are depicted as white, bcps as dark blue and ring critical points as green solid circles.

A gradient trajectory plot of the experimental electron density in the plane subtended by two such nonclassical C─H···O contacts is shown in Figure 6. The significantly curved bond paths associated with the H···O interactions already visually suggest weak contacts. The electron densities in the corresponding bond critical points amount to only 0.05 e·Å^−3^ in Table S4, at the limit of significance; they also confirm that these nonclassical HBs are very weak.

**Figure 7:**
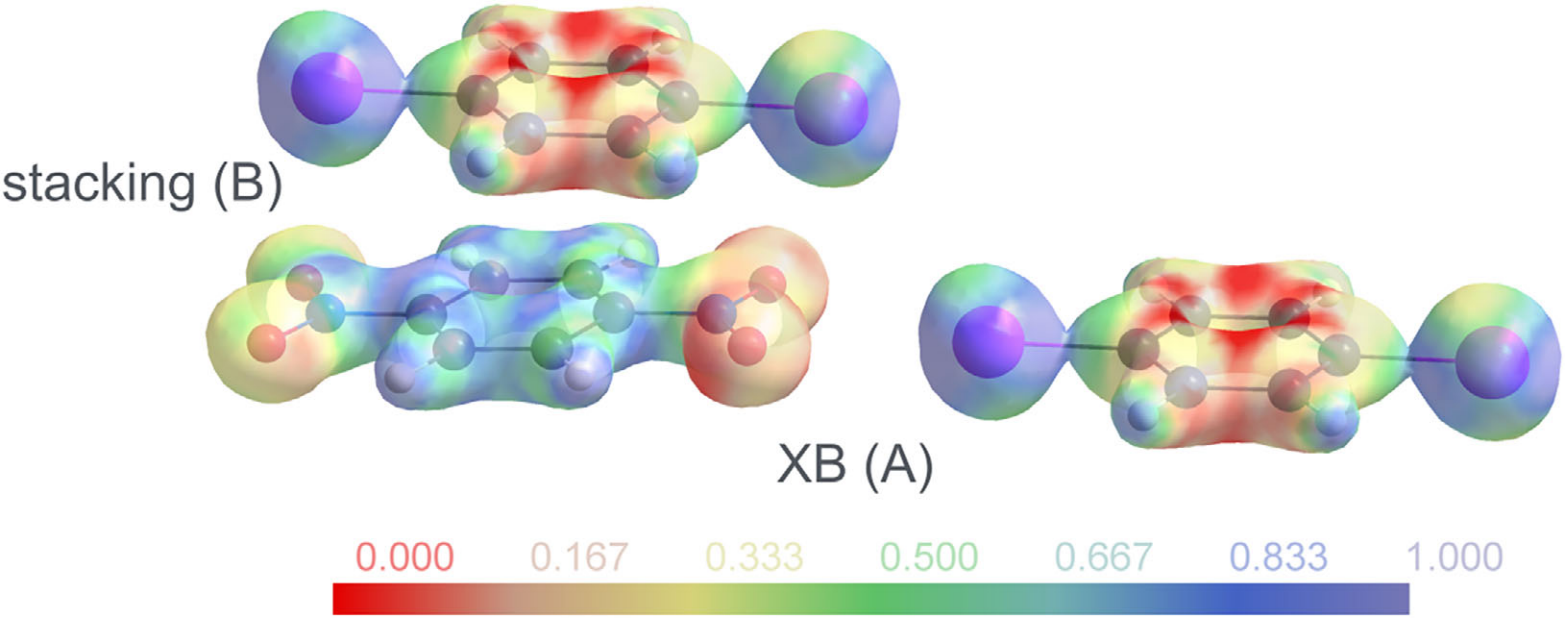
Electrostatic potential mapped on an isosurface of electron density *ρ* = 0.5 e⋅Å^−3^ (program MoleCoolQt^[^ ^35^ ^]^). The interactions correspond to the patterns A and B defined in Figure 5.

Frank Allen's original design principle focused on the idea of a favorable interaction between the nitro group and a neighboring I as potential supramolecular synthons. The experimental results show that both this bifurcated XB and the nonclassical HBs are associated with hardly detectable features of the electron density and can be classified as very weak. The third interaction type also mentioned by Allen et al. refers to stacking of alternative π systems as shown in Figure 5(b). Based on interlayer distances and overlap geometry, the earlier authors assumed that **1**α features “only moderate π···π interactions” but already recognized “that there is some charge transfer”.^[^ ^24^ ^]^ Based on the experimental electron density, the electrostatic potential (ESP) may be assessed. Figure 7 shows the ESP for a dinitrobenzene molecule interacting with two diiodobenzene neighbors in **1**α.

Figure 7 conveys a clear message: Beyond the contact between a nitro group and an iodine atom in the symmetric bifurcated XB (pattern A), stacking of the components in consecutive layers (pattern B) is associated with favorable electrostatic interactions. The above statements concerning electron density were based on high resolution X‐ray diffraction; they necessarily face experimental limits when it comes to very weak interactions. Fortunately, the electron density and its derived properties represent a meeting ground for such experiments and theoretical calculations without any experimental noise. In the following section, we will use the latter to shed light on the directional XBs and HBs as well as on the relevance of interlayer stacking.

### 2.2 Theoretical Calculations

**Figure 8:**
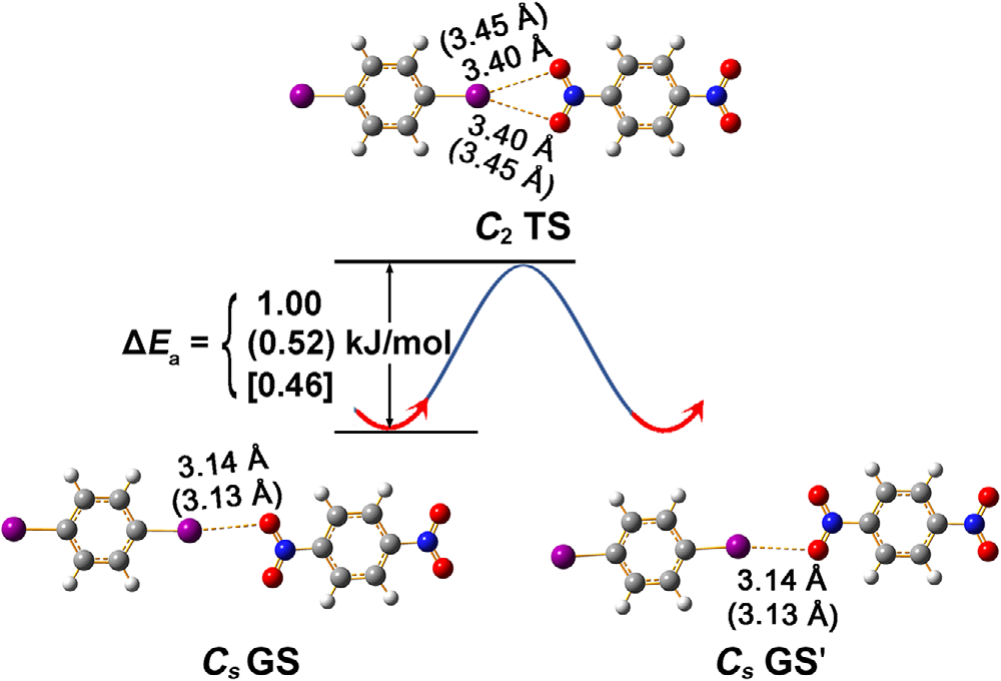
Optimized ground state (GS and GS′) and transition state (TS) structures of (IC_6_H_4_I)(OONC_6_H_4_NOO). I···O distances and energy barrier is indicated at the M06‐2X/6–311 + G(d) level and, in parentheses, at the rev‐VDW‐DF2 level of theory. The corresponding energy barrier at DLPNO‐CCSD(T) is shown in square brackets.

Our first theoretical comparison^[^ ^27^ ^]^ between a linear XB (Figure 8, left and right) and the symmetric bifurcated geometry described by Allen et al.^[^ ^24^ ^]^ (Figure 8, top) was conducted at the molecular level. We found that the symmetric geometry represents the TS between two alternative degenerate ground states GS and GSʹ which correspond to linear XB arrangements. At the M06‐2X level of theory which has proven to be suitable for complex systems with XBs,^[^ ^36^, ^37^, ^38^ ^]^ only a small energy barrier of Δ*E*_a_ = 1.00 kJ/mol is encountered during the reversible fluxional process. Benchmarking calculations have shown that the dispersion‐corrected rev‐VDW‐DF2 approach represents the best choice in describing noncovalent interactions, including halogen and hydrogen bonding in molecular crystals.^[^ ^39^, ^40^, ^41^ ^]^ At this level of theory, the energy barrier is reduced to 0.52 kJ/mol and matches the corresponding value of Δ*E*_a_ = 0.46 kJ/mol obtained at the more accurate DLPNO‐CCSD(T)/def2‐TZVPP level^[^ ^42^, ^43^ ^]^ very well. The discrepancy between a TS at the molecular level and an apparently stable arrangement in the extended crystal induced us to join forces with experimental solid‐state chemists and led to the detection of the structural phase transition.

**Figure 9:**
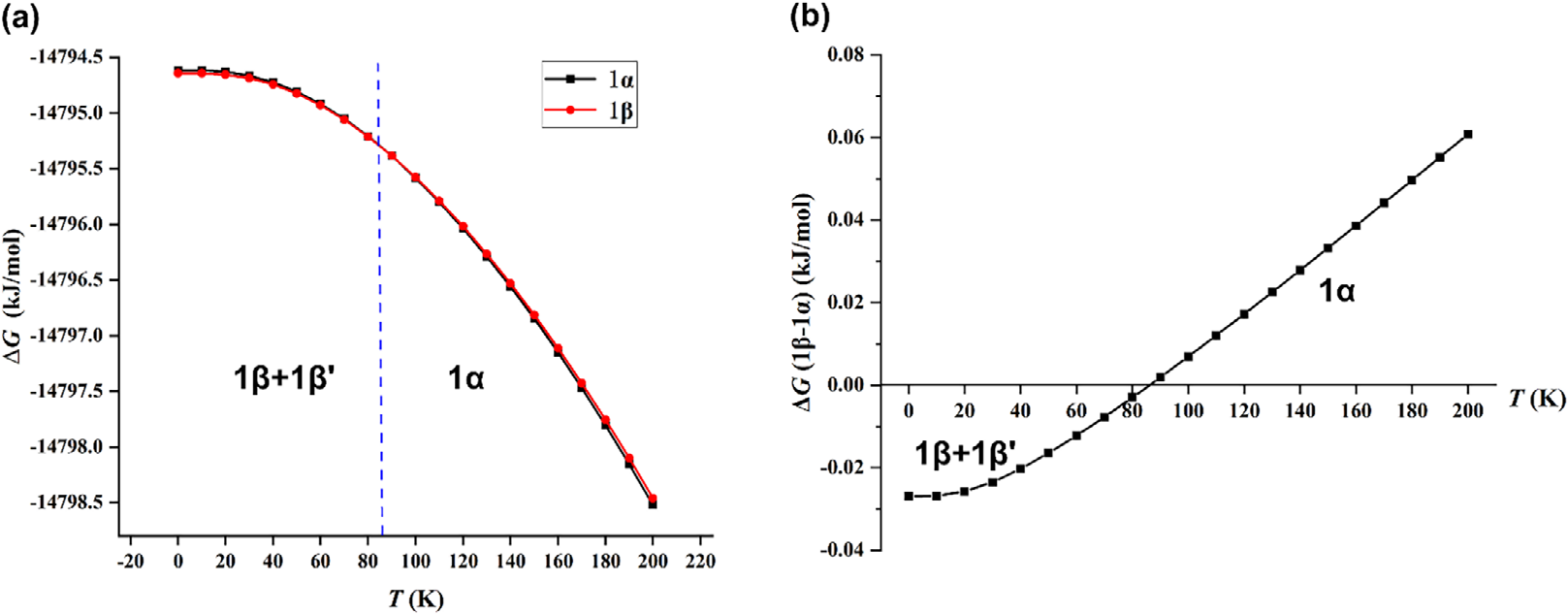
a) Evolution of the Gibbs free energies Δ*G* for the **1**α and **1**β phases with temperature; b) Evolution of the differences of Gibbs free energies Δ*G*(1β−1α) between the **1**α and **1**β phases with temperature.

We therefore proceeded to periodic calculations in the crystals at the rev‐VDW‐DF2 level; a detailed description of the calculation methods has been compiled in the Supporting Information. Entropy effects are taken into account to obtain the Gibbs free energies (Δ*G*) in equation (1) in the Supporting Information. Figure 9(a) shows that the temperature dependence of the Gibbs free energies for **1**α and **1**β are associated with two almost superimposed lines, making it hard to visualize their crossover. For a better visualization and a more accurate definition of the phase transition temperature, the differences in Δ*G* defined as Δ*G*(**1**β−**1**α) = Δ*G*(**1**β)−Δ*G*(**1**α) have been plotted in Figure 9(b) as a function of temperature. Intriguingly, Figure 9(b) shows a crossover between the Gibbs free energies of **1**α and **1**β (Δ*G*(**1**β−**1**α) ≈ 0) around 86 K. Above this calculated phase transition temperature **1**α is slightly more stable and below 86 K, **1**β becomes more favorable. We here emphasize the benefit of a joint approach by experimentalists and theoreticians: experiment tells us that the alternative phases are associated with the same Gibbs energy at the transition temperature. In view of the extremely small energy differences between both phases, the agreement between this calculated transition temperature of 86 K with the experimentally observed phase transition at 130 K is surprisingly good.

In order to facilitate a direct and interaction‐detailed comparison with the experimental results, we performed QTAIM analyses on both **1**α and **1**β crystals. Figure S9 depicts the bond critical points (bcps) corresponding to the XBs and the nonclassical HBs in both phases. Table S5 lists the calculated electron densities at each bcp. The observed effects are small but consistent and allow to perceive a trend. Upon the transition of the fully symmetric **1**α to the slightly asymmetric low temperature phase **1**β, one of the XBs (XB 1 in Figure S9) is strengthened and the other contact (XB 2) becomes weaker; the average electron density of both interactions increases very slightly. In contrast, the average electron density of the 10 weak nonclassical HBs undergoes a small decrease. These results indicate that the overall XB interaction, albeit always weak, is slightly stronger in the asymmetric geometry of **1**β than in the fully symmetric **1**α: XB fluctuation with its low energy barrier can drive the observed reversible phase transition. Even in the absence of systematic errors, experimental work is invariably associated with noise and background. The often‐ quoted standard uncertainties which should accompany every experimentally determined quantity and which are provided for our experimental results represent the somewhat optimistic lower limit for errors. In contrast, such an obvious lower limit does not exist for theoretical calculations. They do not suffer from noise and may, in the absence of systematic errors, even take extremely small interaction energies into account. An inappropriate level of theory can obviously introduce a systematic error; in order to address this aspect, we have performed a series of benchmark calculations at different levels of theory. We summarize our results for the weak directional interactions: The topological analysis of the experimental electron density does not reveal bcps for the bifurcated C─I···O contacts. Theory can detect bcps for these interactions (labeled as contacts 1 and 2 in Figure S9 and Table S5) but consistently finds low values for the associated electron density. A similar situation is encountered for the nonclassical C─H···I HBs; experiment fails to detect bcps, and theory assigns low electron density to these contacts (interactions labeled 3, 5, 8, and 10 in Figure S9 and Table S5). Bond critical points for the (again nonclassical) C─H···O HBs show up both in experiment (Figure 6 and Table S4) and theory (Figure S9 and Table S5) and are even associated with comparable electron densities in the bcps of ca. 0.05 e·Å^−3^. None of the above‐mentioned contacts stands out as a structure‐determining directional interaction. The very weak bifurcated XB corresponds to a fluxional interaction at higher temperature and provides the dynamic mechanism for the reversible phase transition in the crystal.

In contrast to these results on the bifurcated XB and the nonclassical HBs, the contribution of inter‐layer stacking appears to be substantial. To clarify and compare the situation in both phases, two binding patterns in each phase were considered for detailed energy decomposition analyses (EDA) at the M06‐2X/QZ4P level of theory. For the high‐ temperature phase **1**α, these patterns are shown in Figure 5(b); the arrangement in **1**β is very similar. The intra‐layer pattern A refers to the bifurcated XB between 1,4‐diiodobenzene and its XB accepting neighbor depicted in light blue; the interlayer stacking pattern B involving the 1,4‐dinitrobenzene molecule depicted in green is easily recognized in Figure 5(b). The overall interaction energy between the two designated fragments is decomposed as the sum of Pauli repulsion, electrostatic attraction, and orbital interaction according to the equation Δ*E*_int_ = Δ*E*_Pauli_ + Δ*E*_elstat_ + Δ*E*_orb_. Table 1 shows that similar results are obtained for both phases **1**α and **1**β. The interaction energy Δ*E*_int_ associated with the stacking pattern B turns out to be about four times larger than that of the XB binding pattern A. Inter‐layer π–π packing predominates the overall inter‐molecular interactions in both phases with a percentage contribution of about 80%, whereas the symmetric and asymmetric bifurcated XBs contribute only about 20% to the overall intermolecular interaction. This finding provides theoretical support for the experimental phenomenon: the dominant inter‐layer π–π overlap tolerates the phase transition from the symmetrically bifurcated XB arrangement in **1**α to an asymmetric bifurcated XB in **1**β, inducing only marginally small Gibbs free energy changes as indicated in Figure 9.

**Table 1:** EDA analysis of the dimolecular fragments according to patterns A and B at the M06‐2X/QZ4P level; energy values are indicated in kcal·mol^−1^.

	1α		1β	
	Pattern A	Pattern B	Pattern A	Pattern B
Δ*E*_Pauli_	2.39	2.09	2.52	2.16
Δ*E*_elstat_	−2.91	−5.44	−2.96	−5.43
Δ*E*_orb_	−1.17	−3.18	−1.20	−3.19
Δ*E*_int_	−1.68	−6.53	−1.64	−6.46
%	20.5%	79.5%	20.2%	79.8%

These results help to understand the experimental findings. Movement in an extended solid is restricted, and therefore structural phase transitions mostly lead to disintegration of single crystals; we here mention a few exceptions.^[^ ^44^, ^45^, ^46^, ^47^, ^48^, ^49^ ^]^ The apparently directional interactions in cocrystals of 1,4‐diiodobenzene/1,4‐dinitrobenzene have proven to be very weak. The dispersion‐dominated stacking is less directional and tolerates the small fluxional movements of the XBs without significant loss of crystallinity, facilitating the observed reversible **1**α↔**1**β phase transition. The XBs, albeit weak, have an important orientating effect within the layers, although inter‐layer π‐stacking dominates the overall interactions in both crystalline phases **1**α and **1**β. In order to separate π‐stacking and weak XBs, we have conducted full 2D periodic optimizations at the rev‐VDW‐DF2 level of theory; the results are shown in Figure S10. In the absence of π‐stacking, the optimized planar fragment of the **1**β phase with two almost linear XBs per unit cell is about 1.41 kJ/mol more stable than the bifurcated layer of the **1**α phase, consistent with the energy barrier shown in Figure 8. The experimentally observed **1**β phase can thus be perceived as the onset of the fluctuation from bifurcated to linear XBs. The concept of an energetically more favorable low‐symmetry geometry is in line with general considerations of structural phase transitions^[^ ^50^ ^]^ and the symmetry principle.^[^ ^51^ ^]^

## 3 Conclusion

To the extent that crystal structures are regarded as the authoritative answer concerning the spatial arrangement of atoms, phase transitions within crystals can convey information about their dynamics. Allen et al.^[^ ^24^ ^]^ had provided reliable experimental evidence for a fully symmetric bifurcated halogen bond in the solid state, but this structural feature rather corresponds to a weak contact than to a synthon. At the molecular level, this exact arrangement even represents a TS between linear XBs. These messages from diffraction experiments and theory appear contradictory, but we here bridge both aspects: We show that the solid with a symmetric XB (**1**α) undergoes a reversible phase transition to an energetically more favorable asymmetric arrangement (**1**β) at low temperature. The fluxional halogen bond, a phonon in the picture of solid‐state physics, serves as the driving force and provides the dynamic mechanism for the observed reversible phase transition. The new phase may be perceived as the onset of the fluctuation from bifurcated to linear XBs. Diffraction results and quantum chemical calculations agree that directional interactions in both phases are very weak; the less directional inter‐layer stacking is hardly affected by the phase transition and allows for its crystal‐to‐crystal character. In a more general sense, symmetric bifurcated XBs might well represent entropy‐stabilized phases not only for the case reported here but also in chemically different systems. We will investigate their potential transition to energetically more favorable arrangements in experiment and theory.

## Supporting Information

Analytical data and details concerning experiments and theoretical calculations can be found in the Supporting Information. Further details on the crystal structures are available in CIF format. Deposition Numbers < https://www.ccdc.cam.ac.uk/services/structures?id=doi:10.1002/chem.202502514>2478411 (for 1α, multipole refinement), 2478411 (for 1α, independent atom model), and 2478413 (for 1β) contain(s) the supplementary crystallographic data for this paper. These data are provided free of charge by the joint Cambridge Crystallographic Data Centre and Fachinformationszentrum Karlsruhe < http://www.ccdc.cam.ac.uk/structures> Access Structures service. The authors have cited additional references within the Supporting Information.^[^ ^52^, ^53^, ^54^, ^55^, ^56^, ^57^, ^58^, ^59^, ^60^, ^61^, ^62^, ^63^, ^64^, ^65^, ^66^, ^67^, ^68^, ^69^, ^70^, ^71^, ^72^, ^73^, ^74^ ^]^

## Conflict of Interest

The authors declare no conflict of interest.

## Supporting information



Supporting Information

## Data Availability

The data that support the findings of this study are available from the corresponding author upon reasonable request.
