# Supplementary material for: Fluxional Halogen Bonds Versus Interlayer Stacking – Theory Meets Experiment
Source: Chemistry. 2025 Nov 7;31(70):e02514. doi: 10.1002/chem.202502514 (PMC12712753; doi:10.1002/chem.202502514)
Supplement: Supplementary file 1 — Supporting Information [file CHEM-31-e02514-s001.pdf]

Chemistry−A European Journal

Supporting Information

Fluxional Halogen Bonds versus Interlayer Stacking – Theory meets Experiment

Cai Yue Gao, Annika Schmidt, Ruimin Wang, Carsten Strohmann,* Ulli Englert,* and Si-Dian Li*

Supporting Information

Fluxional Halogen Bonds versus Interlayer Stacking – Theory meets Experiment

Cai Yue Gao,[a] Annika Schmidt,[b] Ruimin Wang,[a] Carsten Strohmann,*[b] Ulli Englert,*[c,d] and Si-Dian Li*[a]

[a] Dr. C. Y. Gao, Prof. Dr. R. Wang, Prof. Dr. S.-D. Li
Institute of Molecular Science
Shanxi University
Taiyuan, China, 030006
E-mail: lisidian@sxu.edu.cn

[b] A. Schmidt, Prof. Dr. C. Strohmann
Institute of Inorganic Chemistry
TU Dortmund University
44227 Dortmund, Germany
E-mail: carsten.strohmann@tu-dortmund.de

[c] Prof. Dr. U. Englert
Institute of Inorganic Chemistry
RWTH Aachen University
52056 Aachen, Germany
E-mail: ullrich.englert@ac.rwth-aachen.de

[d] Prof. Dr. U. Englert
Institute of Environmental and Chemical Engineering
Jiangsu University of Science and Technology
Zhenjiang, China, 212003
E-mail: ullrich.englert@ac.rwth-aachen.de

Table of contents

1 Crystallization Experiments 1

1.1 Crystallization from acetone 1

1.2 Crystallization from ethanol 1

2 Diffraction experiments 1

2.1 Data collections 1

2.2 Phase transition 2

2.3 High resolution data completeness 4

2.4 Multipole refinements 6

2.4.1 Experimental electron density study 6

2.4.2 Residual electron density distribution of 1α 9

2.5 Synopsis of crystal data, data collection parameters and refinement results for 1α (independent atom and multipole model) and 1β 11

3 Theoretical Calculations 12

4 Reference 14

1 Crystallization Experiments

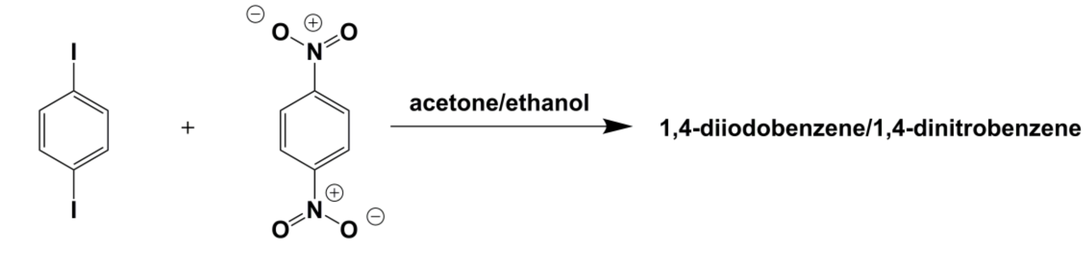

1,4-diiodobenzene 1,4-dinitrobenzene

1.1 Crystallization from acetone

Diiodobenzene (53 mg, 0.16 mmol, 1.00 eq.) and dinitrobenzene (27 mg, 0.16 mmol, 1.00 eq.) were added to a beaker. Acetone (3.00 mL) was added until all solids were dissolved. Upon evaporation of the solvent overnight, yellow crystals of compound 1,4-diiodobenzene/1,4-dinitrobenzene were formed.

1.2 Crystallization from ethanol

Diiodobenzene (7.85 mg, 0.024 mmol, 1.00 eq.) and dinitrobenzene (4.00 mg, 0.024 mmol, 1.00 eq.) were added to a small bottle. Ethanol (3.00 mL) was added until all solids were dissolved. Seal the bottle with a film, and tie several small holes, wait for the solvent to evaporate slowly, yellow crystals of compound 1,4-diiodobenzene/1,4-dinitrobenzene were produced about a month later.

2 Diffraction experiments

2.1 Data collections

Suitable crystals were covered with an inert oil (perfluoroalkylether) and prepared with a *SMZ1279* stereomicroscope from *Nikon Metrology GmbH* and mounted on a *MicroMount* or *MicroLoop* from *MiTeGen*. Crystal structure determination was accomplished on a *Bruker D8 Venture* four-circle diffractometer using a *PHOTON II CPAD* detector by *Bruker AXS GmbH*. X-ray radiation was generated by microfocus source IμS Mo (λ = 0.71073 Å) by *Incoatec GmbH* with HELIOS mirror optics and a single hole collimator by *Bruker AXS GmbH*. For the data collection, the program *APEX5 Suite* (v2023.9-2)[1] with the integrated programs SAINT (integration) and SADABS (adsorption correction) by *Bruker AXS GmbH* were used. The processing and finalization of the crystal structure was done with the program Olex2.[2] The crystal structure

was solved with the ShelXT[3] structure solution program using Intrinsic Phasing and refined with the ShelXL refinement package using Least Squares minimization.[4]

2.2 Phase transition

A more detailed synopsis of the phase transition is provided here.

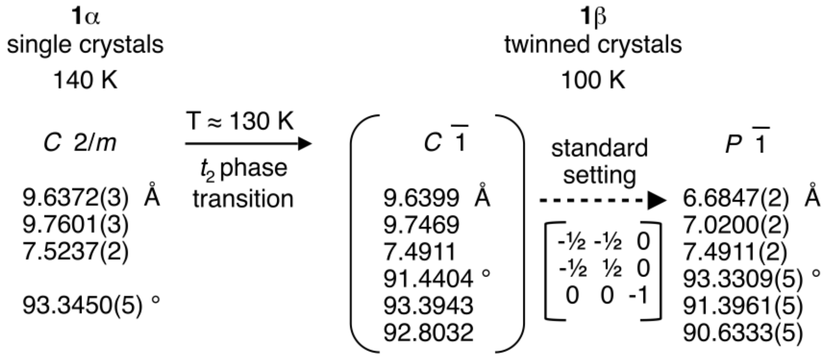

Figure S1 Phase transition and unit cells above and below the phase transition.

Figure S1 gives an overview of the lattice parameters for the high temperature phase **1**α (at 140 K) and the low temperature phase **1**β (at 100 K) and explains their symmetry relationship. Upon cooling, two matrices were required to index the diffraction pattern: the monoclinic *C*-centered cell transforms (solid arrow in Figure S1) into a triclinic cell of very similar dimensions (*translationengleich*). The index of the low temperature subgroup *C*-1 in the high temperature supergroup *C*2/*m* is 2, and therefore the transition can be associated with a t_2 type.[5] In the (unconventional) *C*-1 cell, the twofold symmetry axis lost upon cooling constitutes the twin law relating the domains in **1**β. After transformation, refinement is conducted in the smaller conventional cell and space group *P*-1; the required transformation matrix with determinant 1/2 is given below the dashed arrow in Figure S1.

In addition to the diffraction data collected at high resolution for **1**β (at 100 K) and **1**α (at 140 K), additional intensity data were collected at intermediate temperatures of 110, 120 and 130 K. The data sets collected at 110 and 120 K could be refined with the same structure model derived for **1**β based on the 100 K data. At 130 K, 90% of the reflections could be indexed with a matrix corresponding to the high temperature phase, but less favorable internal agreement between apparently equivalent reflections and significantly higher residuals were encountered. Unaccounted maxima and minima in a final Fourier difference synthesis were substantially higher than for the single crystal data collected at 140 K and for the data collected on twinned crystals at 120,

110 or 100 K. We presume that part of the intensity data were affected by the onset of the phase transition. Figure S2 shows the temperature dependence of the ratio *V/Z, i.e.* the volume per formula unit 1,4-diiodobenzene/1,4-dinitrobenzene.

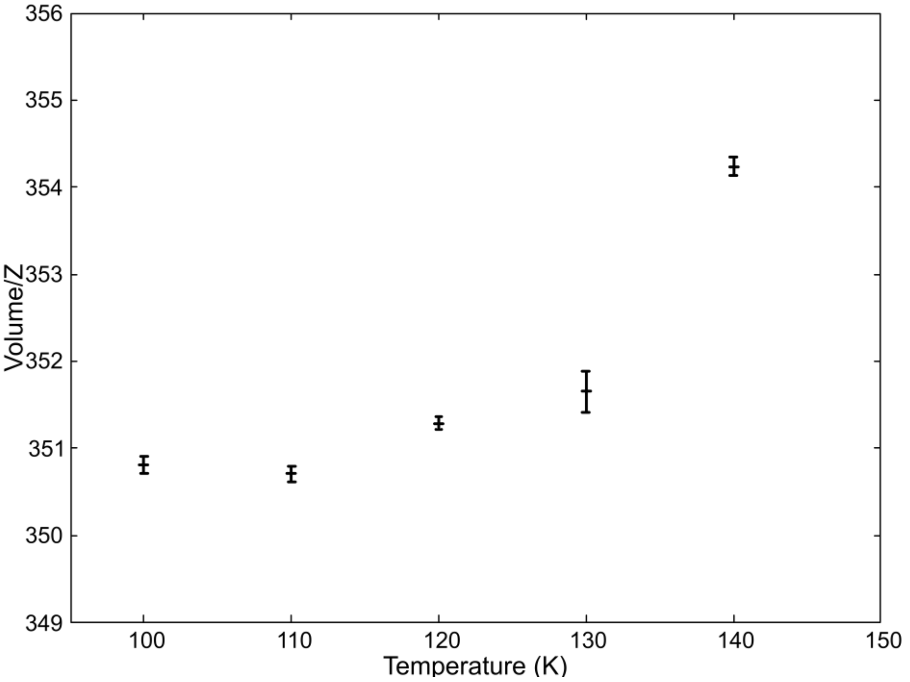

Figure S2 Cell volume per formula unit (*Z*) versus temperature (*T*); the error bars indicate 5 standard uncertainties.

Close to 130 K, the ratio *V/Z* changes; we also note that the standard uncertainty for the unit cell at 130 K is substantially higher than those obtained at the other data collection temperatures. As the phase change occurs close to 130 K, we decided to collect the high resolution data for **1**α at 140 K, at a safe distance above the transition temperature.

2.3 High resolution data completeness

Table S1 Resolution and completeness statistics (cumulative and Friedel pairs averaged) of **1**α.

Theta	sin(th)/Lambda	Complete	Expected	Measured	Total Missing
20.82	0.500	1.000	397	397	0
23.01	0.550	1.000	533	533	0
25.24	0.600	1.000	681	681	0
27.51	0.650	1.000	860	860	0
29.84	0.700	1.000	1086	1086	0
32.21	0.750	1.000	1316	1316	0
34.65	0.800	1.000	1585	1585	0
37.17	0.850	1.000	1907	1907	0
39.77	0.900	1.000	2256	2256	0
42.47	0.950	1.000	2640	2640	0
45.29	1.000	1.000	3078	3078	0
48.27	1.050	1.000	3561	3561	0
51.43	1.100	1.000	4076	4076	0
54.82	1.150	1.000	4658	4658	0
58.53	1.200	1.000	5275	5275	0
62.67	1.250	0.999	5957	5953	4
62.81	1.252	0.998	5982	5970	12

Note: The Reported Completeness refers to the Actual H,K,L Index Range

Table S2 *R*-value statistics as a function of resolution (in resolution shells) of **1**α.

R-Value Statistics as a Function of Resolution (in Resolution Shell)

Theta	sin(Th)/L	#	R1	wR2	S	Rs	av(I/SigW)	av(I)	av(SigW)
12.38	0.302	90	0.019	0.058	2.708	0.005	46.55	12418.28	263.52
15.68	0.380	92	0.012	0.047	2.077	0.004	43.57	6475.33	137.48
18.02	0.435	85	0.009	0.038	1.610	0.004	41.50	4500.33	97.10
19.90	0.479	86	0.010	0.035	1.472	0.004	41.00	3107.79	68.05
21.51	0.516	79	0.011	0.031	1.243	0.004	38.74	2809.18	62.40
22.94	0.548	96	0.010	0.031	1.237	0.005	38.64	2140.21	48.22
24.22	0.577	78	0.011	0.032	1.233	0.005	36.72	1828.91	41.65
25.40	0.603	89	0.012	0.029	1.101	0.006	36.04	1290.30	30.36
26.49	0.628	87	0.011	0.026	0.960	0.007	34.52	1169.26	27.92
27.52	0.650	78	0.013	0.028	1.060	0.007	36.17	1157.74	27.99
28.49	0.671	85	0.013	0.030	0.991	0.009	30.17	797.60	20.41
29.41	0.691	87	0.013	0.028	0.968	0.009	32.07	813.53	20.74
30.28	0.709	83	0.013	0.029	0.928	0.011	29.80	602.83	16.28
31.12	0.727	77	0.012	0.028	0.929	0.011	31.56	638.55	17.03
31.93	0.744	93	0.014	0.032	1.026	0.012	29.67	569.07	15.55
32.71	0.760	92	0.012	0.027	0.814	0.013	27.79	491.02	14.01
33.46	0.776	79	0.013	0.028	0.836	0.014	27.29	410.05	12.08
34.20	0.791	82	0.010	0.023	0.673	0.017	27.23	353.16	11.03
34.91	0.805	70	0.010	0.023	0.667	0.015	26.01	345.93	10.58
62.81	1.252	4362	0.027	0.061	1.035	0.027	13.16	76.01	3.41

R(sig) = sum(sig(I)) / sum(I) = 0.0071

From FCF: R1 = 0.0174(5292), wR2 = 0.0461(5970), S = 1.117
From CIF: R1 = 0.0174(5292), wR2 = 0.0461(5970), S = 1.117, Npar = 52

No (SHELXL) Optimized Weights: wR2 = 0.0319 , S = 2.80

2.4 Multipole refinements

2.4.1 Experimental electron density study

Refinement was conducted with all intensity data $I > 0$. The final refinements on F^2 comprised multipoles up to hexadecapoles for non-H atoms and up to bond directed dipoles for the H atoms. Contraction parameters for non-H atoms; ϰ' was fixed to 3.0 for I, to 1.0 for O, N and C, to 1.2 for H atoms. Contraction parameters and multipole population coefficients have been compiled in Table S3. The positional parameters of H atoms were fixed to subtend a C-H distance of 1.09 Å.

```
Number of data              =   5766
Rejected based on OBS       =      0
Rejected based on SIGOBS    =      0
Rejected based on SINTHL    =      0
Total number of rejections  =      0
Included in the refinement  =   5766


                    Residuals after final cycle

R{ F } =     0.0153                            Rw{ F } =     0.0145
R{F^2} =     0.0126                            Rw{F^2} =     0.0265
 GOFw  =     1.0187          GOF =     2.3043   Nref/Nv =    29.1212
```

Table S3 Monopole populations, radial parameters and net atomic charges.

Atom	Pval	Kappa	Kappa'	Atomic charge
I(1)	6.19(7)	1.250(7)	3.000	+0.81(7)
O(1)	6.34(5)	0.983(5)	1.000	-0.34(5)
N(1)	5.01(11)	0.987(9)	1.000	-0.01(11)
C(1)	4.42(12)	0.967(10)	1.000	-0.42(12)
C(2)	4.22(7)	0.993(7)	1.000	-0.22(7)
C(3)	3.66(13)	1.043(13)	1.000	+0.34(13)
C(4)	4.17(7)	1.002(7)	1.000	-0.17(7)
H(2)	0.82(3)	1.200	1.200	+0.18(3)
H(4)	0.80(3)	1.200	1.200	+0.20(3)

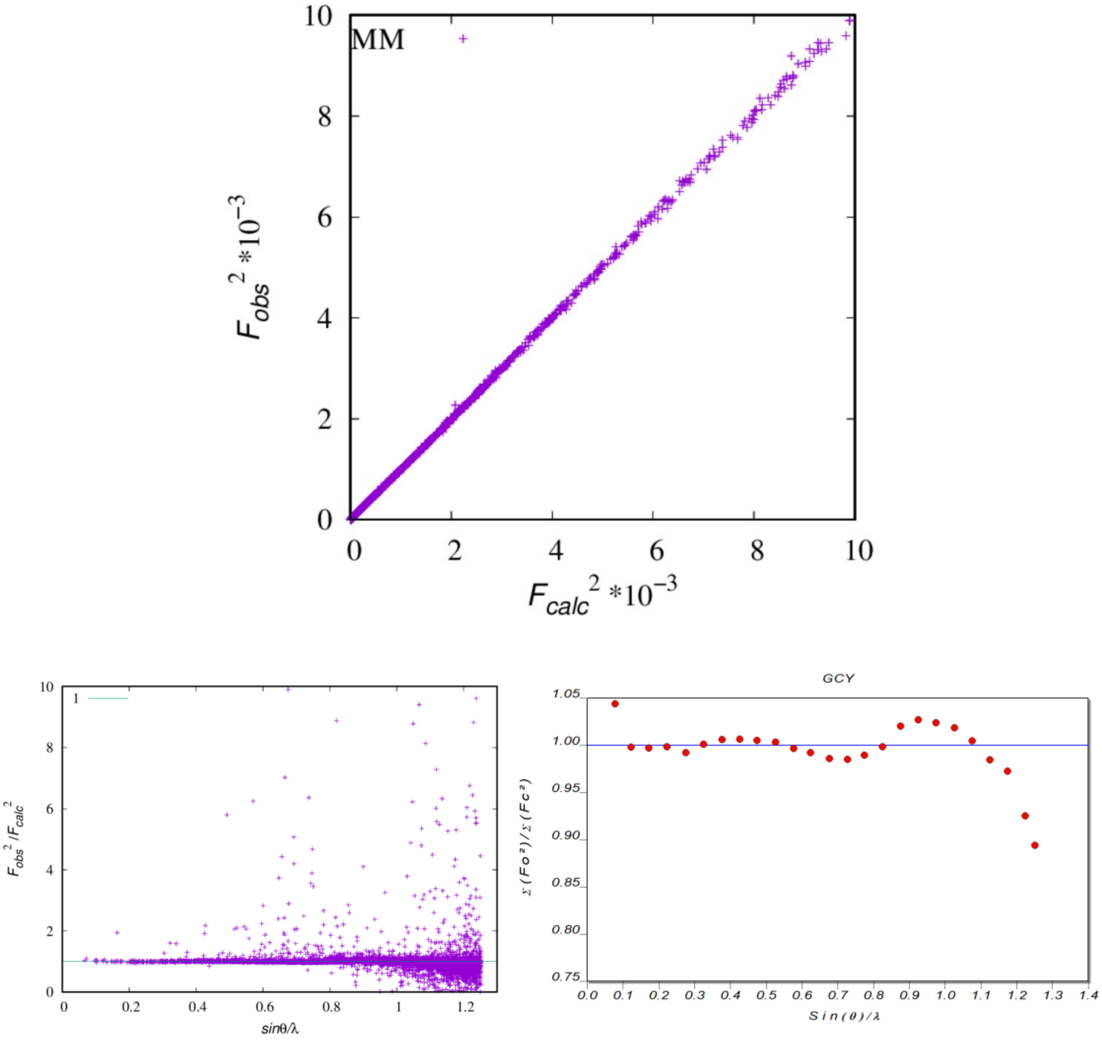

Figure S3 Scatterplots for X-ray refinement result (MM) for **1**α.

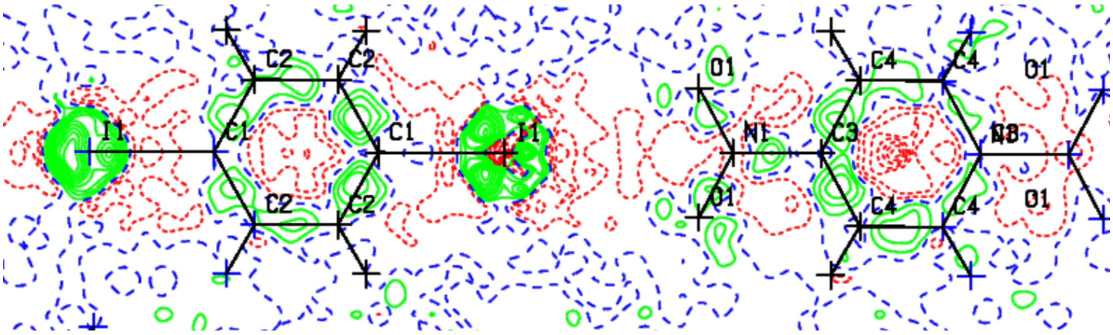

Figure S4 Residual electron density map for **1**α after the IAM refinement. The contour interval is 0.10 e·Å^{-3}, green lines indicate positive, red lines negative and blue lines zero contours.

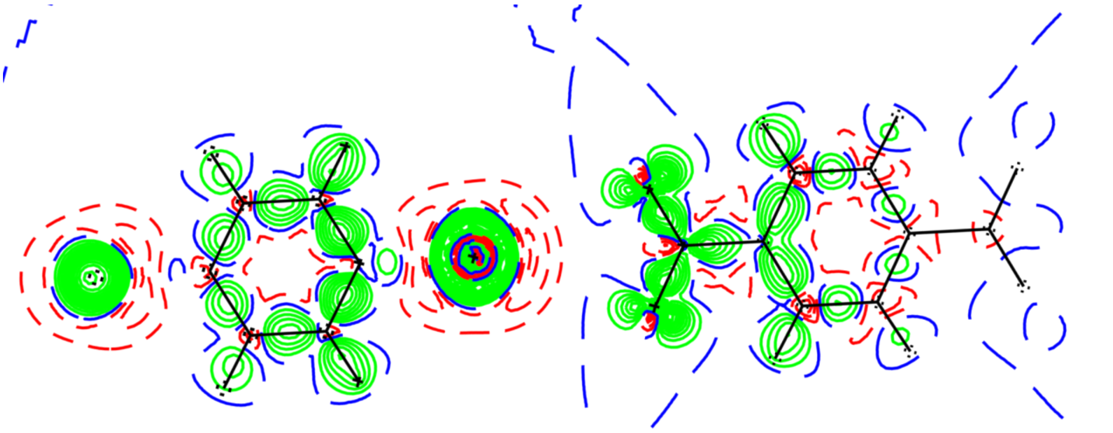

Figure S5 Deformation density map for **1**α after the IAM refinement. The contour interval is 0.10 e·Å^{-3}, green lines indicate positive, red lines negative and blue lines zero contours.

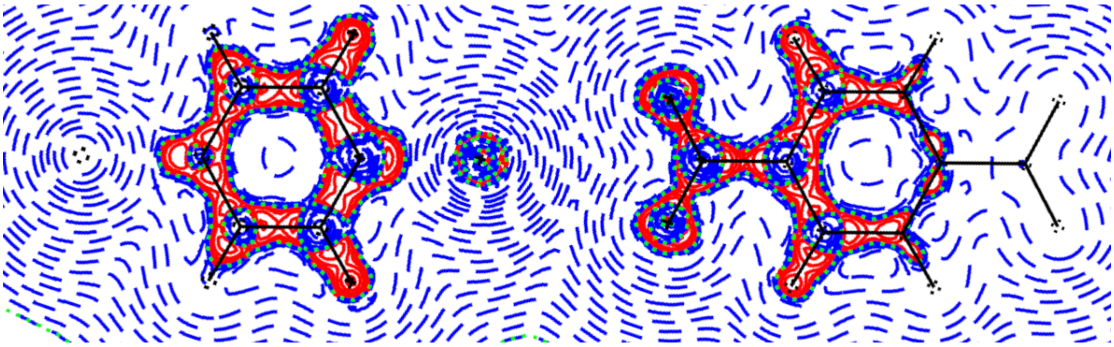

Figure S6 Laplacian of the electron density of **1**α, with positive values in blue, negative values in red and contours at $\pm 2^{n}\cdot 10^{-3}$ e·Å^{-5}.

2.4.2 Residual electron density distribution of 1α

Table S4 Topological properties of interactions and chemical bond at their bond critical point (3, -1). d_1 (d_2) is the distance from the first (second) atom to the (3, -1) critical point, $R_{ij} = d_1 + d_2$, ρ is the electron density, $\nabla^2 p$ is the Laplacian of the electron density. *G* (a.u.) is the kinetic energy density, *G*/*p* (a.u.) the ratio between kinetic energy density and electron density, *V* (a.u.) the potential energy density and *E* (a.u.) the total energy density in the bond critical points (bcps).

Bond	dist.(Å)	R_{ij}(Å)	d_1(Å)	d_2(Å)	$\rho(e{\cdot}Å^{-3})$	$\nabla^2\rho(e{\cdot}Å^{-5})$	$G(a.u.)$	$\frac{G}{\rho}(a.u.)$	$V(a.u.)$	$E(a.u.)$
O1···H2^i	2.49	2.5152	1.4648	1.0504	0.051(5)	0.694(2)	0.0056	0.75	-0.0041	0.0016
O1···H4^{ii}	2.64	2.6654	1.4836	1.1818	0.045(2)	0.569(2)	0.0046	0.69	-0.0033	0.0013
C2^i···C4^{ii}	3.7888(8)	3.7905	1.8540	1.9364	0.017(2)	0.144(2)	0.0011	0.45	-0.0008	0.0004
I1—C1	2.0985(7)	2.0986	1.1529	0.9457	0.70(2)	5.31(3)				
O1—N1	1.2257(11)	1.2258	0.6664	0.5594	3.45(6)	-16.0(2)				
N1—C3	1.4704(10)	1.4703	0.8469	0.6234	1.78(3)	-10.4(2)				
C1—C2	1.3926(6)	1.3928	0.6982	0.6946	2.22(4)	-22.7(2)				
C3—C4	1.3867(6)	1.3867	0.6766	0.7101	2.18(3)	-19.80(8)				

$i = -0.5 + x, -0.5 + y, z$; $ii = 0.5 - x, -0.5 - y, -z$

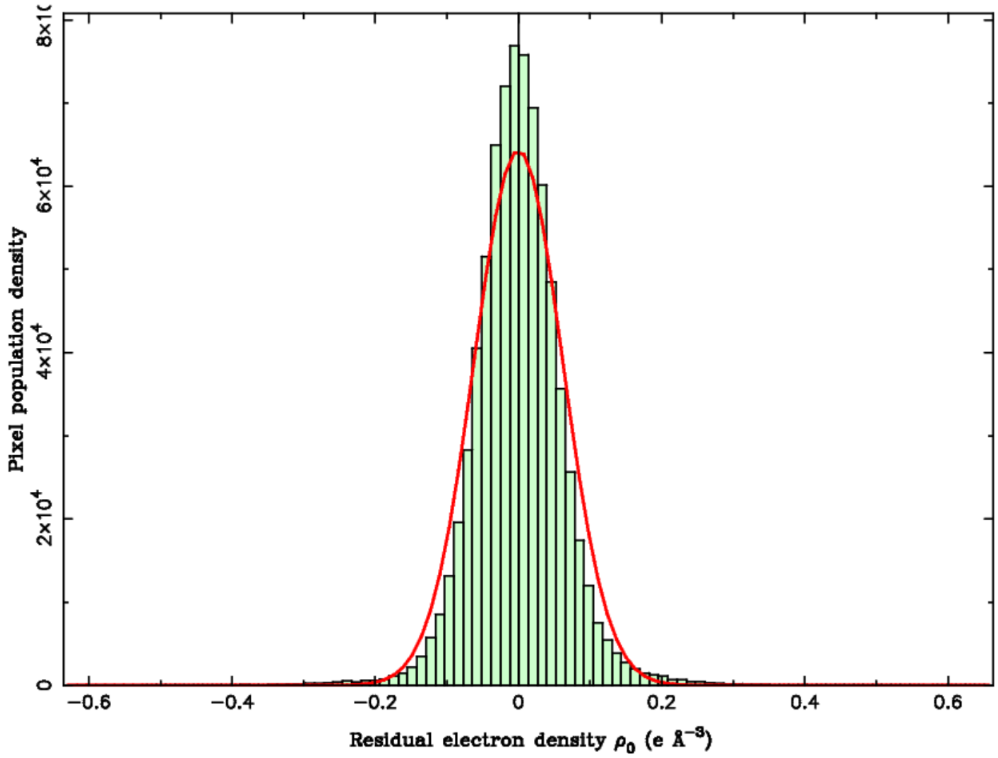

Figure S7 Probability distribution histogram of residual electron density of **1**α.

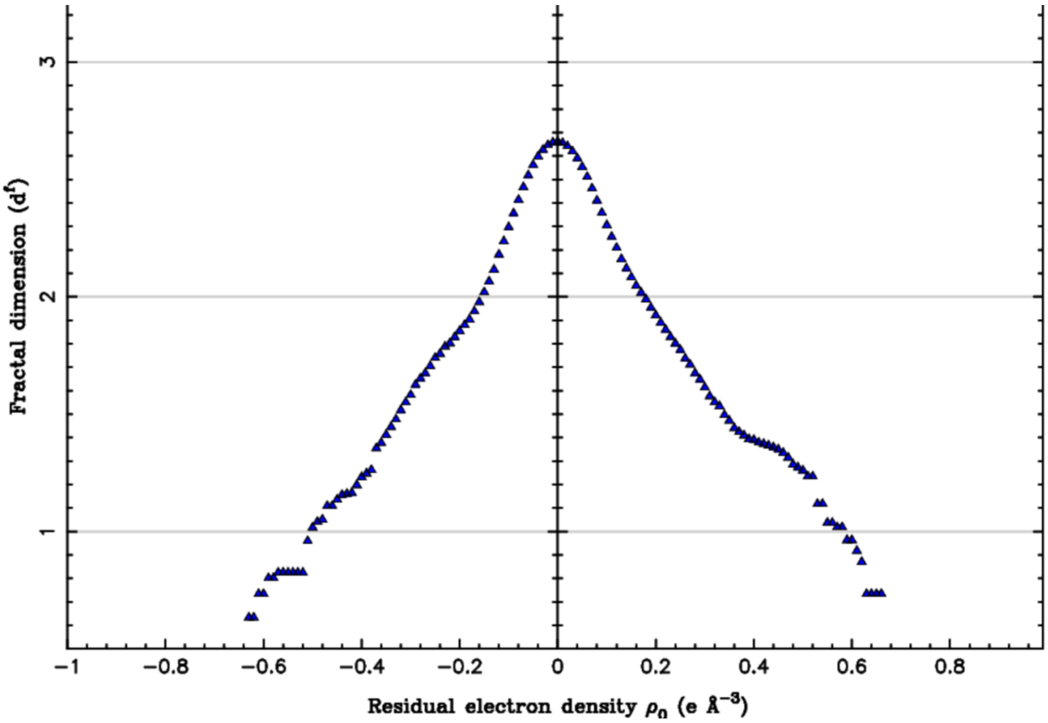

Figure S8 Fractal dimension plot of residual electron density of **1**α.

2.5 Synopsis of crystal data, data collection parameters and refinement results for 1α (independent atom and multipole model) and 1β

**Compound**	**1**α (IAM)	**1**α (MM)	**1**β
Empirical formula	$C_{12}H_8I_2N_2O_4$		$C_{12}H_8N_2O_4I_2$
Formula weight	498.00		498.00
Temperature/K	140(2)		100(2)
Crystal system	monoclinic		triclinic
Space group	*C*2/*m*		$P\bar{1}$
a/Å	9.6372(3)		6.6847(2)
b/Å	9.7601(3)		7.0200(2)
c/Å	7.5237(2)		7.4911(2)
α/°	90		93.3309(5)
β/°	93.3450(5)		91.3961(5)
γ/°	90		90.6333(5)
Volume/Å^3	706.47(4)		350.806(17)
Z	2		1
ρ_{calc}g/cm^3	2.341		2.357
μ/mm^{-1}	4.465		4.496
F(000)	464.0		232.0
Crystal size/mm^3	0.209 × 0.195 × 0.116		0.209 × 0.195 × 0.116
Radiation	MoK*α* (*λ* = 0.71073)		MoK*α* (*λ* = 0.71073)
2*Θ* range for data collection/°	5.424 to 125.622		5.448 to 124.862
Index ranges	-24 ≤ *h* ≤ 23, -24 ≤ *k* ≤ 24, -18 ≤ *l* ≤ 18		-15 ≤ *h* ≤ 16, -16 ≤ *k* ≤ 16, -16 ≤ *l* ≤ 16
Reflections collected	260892		114284
Independent reflections	5970 [R_{int} = 0.0481, R_{sigma} = 0.0071]		15832 [R_{int} = 0.0502, R_{sigma} = 0.0241]
Data/restraints/parameters	5970/0/52	5766/0/198	15832/0/92
Goodness-of-fit on F^2	1.117	1.019	1.061
Final *R* indexes [I>=2σ (I)]	R_1 = 0.0174, wR_2 = 0.0437	R_1 = 0.0144	R_1 = 0.0368, wR_2 = 0.0761
Final *R* indexes [all data]	R_1 = 0.0210, wR_2 = 0.0461	R_1 = 0.0153, wR_2 = 0.0265	R_1 = 0.0444, wR_2 = 0.0811
Largest diff. peak/hole / e·Å^{-3}	1.24/-0.86	0.66/-0.64	4.09/-1.06

3 Theoretical Calculations

Structural optimizations on crystals were carried out using the Vienna ab initio simulation package (VASP 5.4)[6, 7] within the rev-vdW-DF2 functional[8] and projector-augmented wave (PAW) potential.[9,10] The rev-vdW-DF2 functional is a revised version of the vdW-DF2 functional[11] with high accuracy in describing van der Waals interactions. The rev-vdW-DF2 functional has been shown to be the best choice for describing non-covalent interactions in molecular crystals.[12] The kinetic energy cutoff of the basis set is 500 eV, the Brillouin zones were sampled using the Monkhorst-Pack method at a spacing of 2π × 0.02 $Å^{-1}$.[13] The convergence of energy during structural relaxation was set to 10^{-5} eV, and the convergence of force on each atom was set to 10^{-3} eV·$Å^{-1}$. The free energy was calculated using the Phonopy program,[14] as follows:

$$\Delta G=\Delta U+\frac{1}{2}\sum_{qv}\hbar\omega_{qv}+k_B T\sum_{qv} ln\left[1-exp\left(-\hbar\,\omega_{qv}/k_B\,T\right)\right] \qquad (1)$$

where ΔU is the energy of the ground state, k_B is the Boltzmann constant, T is the temperature, and ω_{qv} is the phonon frequency for the band v at wave vector q.

For the alternative arrangements of (IC_6H_4I)($OONC_6H_4NOO$) and the energy barrier in Figure 8, single-point relative energies were further refined with the more accurate method (DLPNO-CCSD(T))[15] implemented in the ORCA 5.0.3 package[16], with the def2-TZVPP basis set[17] at the M06-2X optimized geometries.

Energy decomposition analysis (EDA)[18] was performed using the ADF program[19] at the M06-2X/QZ4P level[20], and the zero-order regular approximation[21-23] was used to explain the scalar relativistic effect. In the EDA analysis, the interaction energy (ΔE_{int}) between two fragments is decomposed into the Pauli repulsion (ΔE_{Pauli}), electrostatic interaction energy (ΔE_{elstat}), and orbital interaction energy (ΔE_{orb}), as shown in equation (2):

$$\Delta E_{int} = \Delta E_{Pauli} + \Delta E_{elstat} + \Delta E_{orb} \qquad (2)$$

QTAIM analysis[24,25] in crystals was performed using the program Multiwfn[26] and visualized by the program VMD[27].

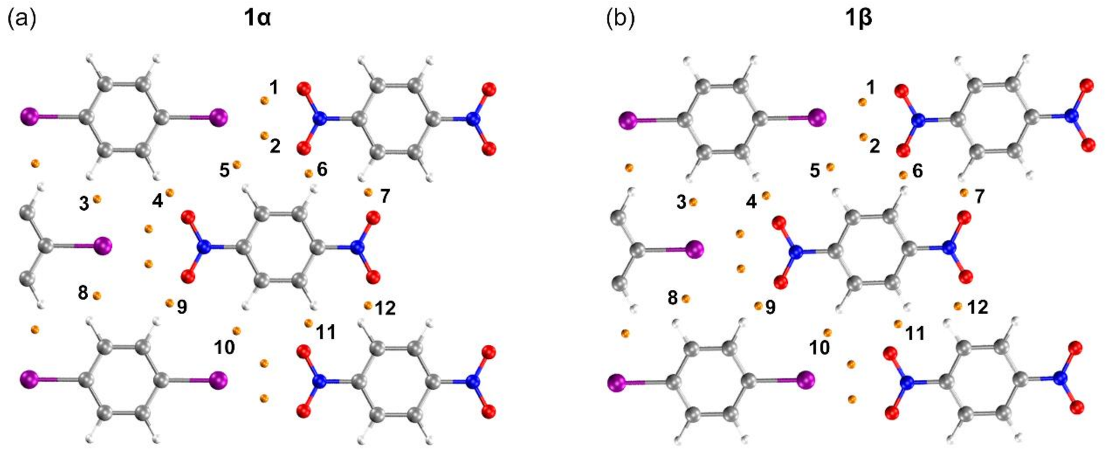

Figure S9. QTAIM analyses of (a) **1**α and (b) **1**β. The orange spheres represent bond critical points, with labels 1 and 2 representing the bcps corresponding to the C-I···O XBs, while labels 3-12 correspond to the C-H···O and C-H···I HBs.

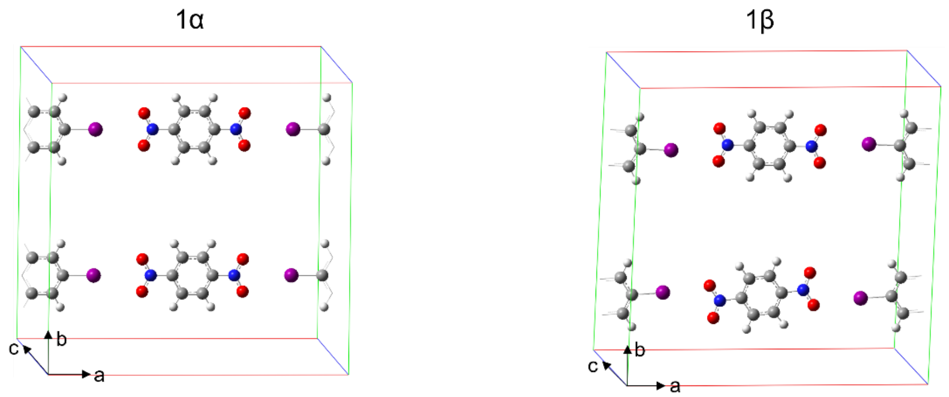

	1α	1β
	*P*2/*m*	*P*1
	a = 20.4443Å, b = 19.8860Å, c = 19.5734Å	a = 20.3797Å, b = 20.1757Å, c = 19.5595Å
	α = 90°, β = 89. 7963°, γ = 90°	α = 90.0312°, β = 89.9346°, γ = 88.0319°
Δ*E*/ kJ/mol	0.00	-1.41

Figure S10. Comparison of the optimized halogen bonds in **1**α and in **1**β phases in the absence of π-packing, with the lattice parameters and relative energies ΔE indicated. We here report the direct optimization results; the 1α optimized structure may be converted to a standard setting (β > 90°). The structure of 1β almost adopts local (non-crystallographic) inversion with respect to the centroids of the dinitrobenzene molecules. The non-standard setting of the lattice parameters for 1β has not been changed to show the close relationship between the phases.

Table S5. Values of electron density (*ρ*) at bcps corresponding to halogen bonds (XBs) and hydrogen bonds (HBs) in **1**α and **1**β crystals, in units of e·Å^{-3}.

	Interaction label	**1**α	**1**β
XBs	1	0.0513	0.0580
	2	0.0513	0.0466
	in average	0.0513	0.0523
HBs	3	0.0378	0.0398
	4	0.0574	0.0594
	5	0.0358	0.0358
	6	0.0472	0.0526
	7	0.0472	0.0526
	8	0.0378	0.0364
	9	0.0574	0.0526
	10	0.0358	0.0337
	11	0.0472	0.0418
	12	0.0472	0.0418
	in average	0.0452	0.0445

4 Reference

[1] Bruker, *Apex5*, Bruker AXS Inc., Madison, Wisconsin, USA **2023**.

[2] O. V. Dolomanov, L. J. Bourhis, R. J. Gildea, J. A. K. Howard, H. Puschmann, *OLEX2*: A Complete Structure Solution, Refinement and Analysis Program. *J. Appl. Cryst.* **2009**, *42*, 339-341.

[3] G. M. Sheldrick, *SHELXT*–Integrated Space-Group and Crystal-Structure Determination. *Acta Cryst.* **2015**, *A71*, 3-8.

[4] G. M. Sheldrick, Crystal Structure Refinement with *SHELXL*. *Acta Cryst.* **2015**, *C71*, 3-8.

[5] U. Müller, Symmetry Relationships between Crystal Structures: Applications of Crystallographic Group Theory in Crystal Chemistry, Oxford University Press, Oxford, **2013**.

[6] G. Kresse, J. Furthmüller, Efficiency of *ab-initio* Total Energy Calculations for Metals and Semiconductors using a Plane-Wave Basis Set. *Comput. Mater. Sci.* **1996**, *6*, 15-50.

[7] G. Kresse, J. Furthmüller, Efficient Iterative Schemes for *ab initio* Total-Energy Calculations using a Plane-Wave Basis Set. *Phys. Rev. B* **1996**, *54*, 11169-11186.

[8] I. Hamada, van der Waals Density Functional made Accurate. *Phys. Rev. B* **2014**, *89*, 121103.

[9] P. E. Blöchl, Projector Augmented-Wave Method. *Phys. Rev. B* **1994**, *50*, 17953.

[10] G. Kresse, D. Joubert, From Ultrasoft Pseudopotentials to the Projector Augmented-Wave Method. *Phys. Rev. B* **1999**, *59*, 1758.

[11] K. Lee, É. D. Murray, L. Z. Kong, B. I. Lundqvist, D. C. Langreth, Higher-Accuracy van der Waals Density Functional. *Phys. Rev. B Condens. Matter* **2010**, *82*, 081101.

[12] F. Tran, L. Kalantari, B. Traoré, X. Rocquefelte, P. Blaha, Nonlocal van der Waals Functionals for Solids: Choosing an Appropriate One. *Phys. Rev. Mater.* **2019**, *3*, 063602.

[13] H. J. Monkhorst, J. D. Pack, Special Points for Brillouin-zone Integrations. *Phys. Rev. B* **1976**, *13*, 5188.

[14] A. Togo, F. Oba, I. Tanaka, First-Principles Calculations of the Ferroelastic Transition between Rutile-type and $CaCl_2$-type SiO_2 at High Pressures. *Phys. Rev. B Condens. Matter* **2008**, *78*, 134106.

[15] Y. Guo, C. Riplinger, U. Becker, D. G. Liakos, Y. Minenkov, L. Cavallo, F. Neese, Communication: An Improved Linear Scaling Perturbative Triples Correction for The Domain Based Local Pair Natural Orbital Based Singles and Doubles Coupled Cluster Method [DLPNO-CCSD(T)]. *J. Chem. Phys.* **2018**, *148,* 011101.

[16] F. Neese, Software Update: The ORCA Program System—Version 5.0. Wiley Interdiscip. *Rev. Comput. Mol. Sci.* **2022**, *12*, e1606

[17] F. Weigend, R. Ahlrichs, Balanced Basis Sets of Split Valence, Triple Zeta Valence and Quadruple Zeta Valence Quality for H to Rn: Design and Assessment of aAccuracy**.** *Phys. Chem. Chem. Phys.* **2005**, *7*, 3297-3305.

[18] M. P. Mitoraj, A. Michalak, T. Ziegler, A Combined Charge and Energy Decomposition Scheme for Bond Analysis. *J. Chem. Theory Comput.* **2009**, *5*, 962-975.

[19] G. Te Velde, F. M. Bickelhaupt, E. J. Baerends, C. Fonseca Guerra, S. J. A. Van Gisbergen, J. G. Snijders, T. Ziegler, Chemistry with ADF. *J. Comput. Chem.* **2001**, *22*, 931-967.

[20] Y. Zhao, D. G. Truhlar, The M06 Suite of Density Functionals for Main Group Thermochemistry, Thermochemical Kinetics, Noncovalent

Interactions, Excited States, and Transition Elements: Two New Functionals and Systematic Testing of Four M06-class Functionals and 12 other Functionals. *Theor. Chem. Acc.* **2008**, *120*, 215-241.
[21] C. Chang, M. Pelissier, P. Durand, Regular Two-Component Pauli-like Effective Hamiltonians in Dirac Theory. *Phys. Scr.* **1986**, *34*, 394.
[22] J. Heully, I. Lindgren, E. Lindroth, S. Lundqvist, Diagonalisation of the Dirac Hamiltonian as a Basis for a Relativistic Many-body Procedure. *J. Phys. B* **1986**, *19*, 2799.
[23] E. v. Lenthe, E. J. Baerends, J. G. Snijders, Relativistic Regular Two-Component Hamiltonians. *J. Chem. Phys.* **1993**, *99*, 4597-4610.
[24] R. F. W. Bader, G. A. Jones, Electron-Density Distributions in Hydride Molecules. The Ammonia Molecule. *J. Chem. Phys.* **1963**, *38*, 2791-2802.
[25] R. F. W. Bader, A Quantum Theory of Molecular Structure and Its Applications. *Chem. Rev.* **1991**, *91*, 893-928.
[26] T. Lu, F. W. Chen, Multiwfn: A Multifunctional Wavefunction Analyzer. *J. Comput. Chem.* **2012**, *33*, 580-592.
[27] W. Humphrey, A. Dalke, K. Schulten, VMD: Visual Molecular Dynamics. *J. Mol. Graph.* **1996**, *14*, 33-38.
